# Supplementary material for: The Polysomnographical Meaning of Changed Sleep Quality—A Study of Treatment with Reduced Time in Bed
Source: Brain Sci. 2023 Oct 7;13(10):1426. doi: 10.3390/brainsci13101426 (PMC10605173; doi:10.3390/brainsci13101426)
Supplement: Supplementary file 1 [file brainsci-13-01426-s001.zip › brainsci-2614265-supplementary.pdf]

Supplementary

Table S1. Correlations between subjective and objective variables at baseline and at treatment week 1. First half of PSG variables

	TIB	WA-SO	TST	Sleep lat.	Sleep eff	#aw
**Baseline**						
**Diff asleep**	.01	-.02	.16	-.43*	.23	-.01
**Sleep qual**	-.24	-.37*	.14	-.14	.38*	-.13
**Dist sleep**	.16	.23	.02	.16	-.21	.32
**Early aw**	-.25	.23	-.01	-.09	.10	.10
**# aw**	-.01	.14	.27	-.22	-.41*	-.41*
**Enough sl**	-.25	.31	.02	-.14	.31	-.20
**Well rested**	.03	.20	.44*	-.25	.52**	-.31
**Ease aw**	-.07	.03	-34°	-.37*	.47**	-.24
**SQindex**	-.16	-.12	.11	.21	-.27	.16
**RSindex**	-.10	-.45*	.37*	-.17	-.02	.15
**ISI**	-.09	.10	-.21	.11	-.14	-.04
**Treatment week 5**						
**Diff asleep**	-.02	-.20	.08	-.11	.21	-.13
**Sleep qual**	-.01	-.13	.01	.35°	.04	-.01
**Dist sleep**	-.13	-.08	-.12	.30	-.07	.20
**Early aw**	.23	-.11	.17	.25	-.09	.09
**# aw**	.14	-.29	.14	-.20	.07	-.40*
**Enough sl**	.31	-.30	.29	-.28	.07	-.15
**Well rested**	20	-.39*	.21	.12	.09	-.09
**Ease aw**	.03	-.37*	.10	.09	.22	-.24
**SQindex**	-.21	-.24	-.27	.22	.04	-.03
**RSindex**	-.36*	-.09	-.06	.12	.02	-.17
**ISIindex**	-.09	-.35*	-.01	-.26	.36*	.20

*=p<.05, **=p<.01. SQ = Sleep quality index. RS = Restorative sleep index. ISI = Insomnia Severity Scale. # aw = number of awakenings. sl=sleep.

Table S2. Correlations between subjective and objective variables at baseline and at treatment week B) Second half of PSG variables

	REM %	N1 %	N2 %	N3 %	REM min	N1 min	N2 min	N3 min
**Baseline**								
**Diff asleep**	-.26	-.10	.12	.19	-.10	-.03	.22	.17
**Sleep qual**	-.05	-.06	.03	.05	.07	-.09	.17	.10
**Dist sleep**	-.28	.10	-.05	.17	-.15	.10	-.03	.09
**Early aw**	-.01	.15	-.18	.06	.03	.08	-.08	.09
**# aw**	.27	-.26	.20	-.17	.31	-.17	.33	-.13
**Enough sl**	-.39*	-.09	-.03	-.18	.28	-.10	.06	-.05
**Well rested**	-.37*	-.30	.23	-.25	.45*	-.16	.48**	-.17
**Ease aw**	.34	-.28	.10	-.07	.40*	-.15	.31	-.03
**SQindex**	-.22	.05	-.05	.20	-.05	.04	,10	.14
**RSindex**	-46**	-.30	,15	-.22	.48**	-.18	.39*	.12
**ISI**	-.25	.03	.02	.17	-.31	-.03	-.14	.06
**Treatment week 5**								
**Diff asleep**	.16	-.16	-.04	.18	.16	-.08	-.05	.20
**Sleep qual**	-.13	.00	.00	.09	.00	.02	-.03	.11
**Dist sleep**	-.01	.11	-.04	-.11	-.05	.06	-.13	-.08
**Early aw**	-.38*	.31	.04	-.24	-.19	.35*	.12	-.12
**# aw**	-.25	-.26	.32	.12	-.12	.21	.36*	.10
**Enough sl**	.00	-.03	.08	-.16	.12	.13	.24	-.06
**Well rested**	-.14	-.21	.17	.04	.20	-.14	.26	-.03
**Ease aw**	.20	-.30	.06	.22	.18	-.24	.01	.20
**SQindex**	-.18	.13	-.10	-.10	-.08	.04	.-,25	.22
**RSindex**	.08	-.13	.13	-.14	.21	-.08	.03	-.16
**ISIindex**	.18	.28	-.36*	.04	.03	,03	.18	.07

*=p<.05, **=p<.01. SQ = Sleep quality index. RS = Restorative sleep index. ISI = Insomnia Severity Scale. # aw = number of awakenings. sl=sleep.
